# Predictors of Left Ventricular Outflow Tract Obstruction After Conventional Repair for Patients with Interrupted Aortic Arch or Coarctation of the Aorta, Combined with Ventricular Septal Defect: A Single-Center Experience

**DOI:** 10.1007/s00246-021-02749-0

**Published:** 2021-10-26

**Authors:** Katarzyna Szaflik, Sebastian Goreczny, Katarzyna Ostrowska, Piotr Kazmierczak, Maciej Moll, Jadwiga A. Moll

**Affiliations:** 1 Department of Cardiology, Polish Mother’s Memorial Hospital, Research Institute, Rzgowska 281/289, 98-338 Lodz, Poland; 2 Department of Cardiac Surgery, Polish Mother’s Memorial Hospital, Research Institute, Lodz, Poland

**Keywords:** Left ventricular outflow tract obstruction, Interrupted aortic arch, Coarctation of the aorta, Aortic valve

## Abstract

Left ventricular outflow tract obstruction (LVOTO) affects survival and reoperation rates after surgical treatment of patients with interruption of the aortic arch (IAA) or coarctation of the aorta (CoA) with ventricular septal defect (VSD). The aim of the study was to determine predictors of LVOTO and to evaluate the relationship between aortic valve (AoV) morphology and the re-intervention rate. Retrospective review of patients, who underwent a conventional repair for IAA/CoA with VSD at a tertiary referral center between 1996 and 2017. The preoperative demographic data as well as pre- and post-operative echocardiographic parameters and re-interventions were reviewed. In the median follow-up of 8.3 years (range of 6.15–10.27) 5 patients (11.9%) from a total of 47 patients included in the study presented with a significant LVOTO. Four of them required reoperation after median period of 2.3 years (range of 0.3–7.9) after the initial surgery. Multivariable logistic regression identified AoV z-score (OR 0.44, *p* = 0.017) as predictor of LVOTO. The mean AoV z-score before the primary repair was significantly smaller in those with LVOTO as compared to those with unobstructed flow from the LV (− 3.58 ± 1.96 vs. − 1.44 ± 1.55; *p* = 0.0016). At 1-year follow-up, both groups showed an increase in the AoV z-score (*p* = 0.98). The re-intervention rate after primary repair (both surgical procedures and percutaneous interventions), either for LVOTO or reCoA, was higher in patients with AoV z-score ≤  − 3 (*p* = 0.007 vs. *p* = 0.46) and those, whose aortic annulus was less or equal than patient’s weight (kg) + 1.5 mm as compared to those with larger aortic annulus (*p* = 0.03 vs. *p* = 0.16). In patients after surgical treatment of IAA/CoA with VSD, the AoV z-score at diagnosis is a significant risk factor for reoperation for LVOTO. With age, AoV growth and z-score improvement is expected. Small AoV at diagnosis is correlated with increased rate of re-intervention for LVOTO and reCoA.

**Supplementary Information:**

The online version contains supplementary material available at 10.1007/s00246-021-02749-0.

## Introduction

The surgical treatment of congenital abnormalities of the aorta, such as interrupted aortic arch (IAA) and coarctation of the aorta (CoA) combined with ventricular septal defect (VSD), has made great advances in the recent years [1, 2]. Various degrees of left ventricular outflow tract obstruction (LVOTO) such as small aortic valve (AoV) or subaortic stenosis are often associated with IAA/CoA with VSD. LVOTO is an important factor affecting survival and reoperation rates after surgical treatment of patients with IAA/CoA with VSD; however, parameters predicting the likelihood of postoperative LVOTO remain controversial [3, 4]. The aim of the study was to determine predictors of LVOTO after the repair of IAA/CoA with VSD and to evaluate the relationship between AoV morphology and the re-intervention rate.

## Materials and Methods

In this retrospective case review study all patients who underwent a conventional repair for IAA/CoA with VSD at a tertiary referral center between 1996 and 2017 were included. The preoperative demographic data (age, weight, height, body surface area) as well as echocardiographic recordings were reviewed at the time of diagnosis and at follow-up visits. The echocardiographic recordings were reviewed in order to determine: morphology of the AoV (bicuspid or tricuspid); diameter and z-score, mitral valve (MV) diameter and z-score, left ventricular (LV) dimensions and function. Documentation from follow-up inpatient and outpatient admissions were reviewed in search of re-operations and percutaneous interventions performed after the conventional repair for IAA/CoA with VSD.

The patients were divided in two groups according to the presence of LVOTO (valvar, subvalvar, and supravalvar obstruction) in follow-up. The growth rate of the AoV and the need for surgical and trans-catheter re-interventions were compared between the two groups. Further, the surgical outcomes after primary repair were analyzed with regard to AoV z-score (AoV >  − 3 and AoV ≤  − 3) and AoV annulus index introduced by Hirata et al. In the latter case, AoV annulus greater than the patient’s weight (kg) + 1.5 mm was used as a cut off value. The z-score was defined as the degree of standard deviation (SD) away from the mean diameter for a normal population, as indexed to the body surface area. Petersen’s method was used for calculation of AV z-score [5].

### Statistical Analysis

Normality of continuous variables was tested using Shapiro–Wilk test. Depending on the normality of the continuous variables, parametric or non-parametric methods were applied in the analysis. Differences in continuous variables between groups were tested using *t*-Student’s test or *U* Mann–Whitney test for two groups, and using ANOVA with Fisher’s Least Significant Difference (LSD) post hoc, Kruskal–Wallis with Dunn post hoc test or Mood’s median test for more groups, based on number of observations. For nominal data Fisher’s exact test or Chi^2^ test with Yates’ correction for continuity was used, based on the number of observations. For evaluation of change in AoV z-score between time points repeated measures ANOVA with Fisher’s LSD post hoc was applied. Impact of continuous and categorical variables on LVOTO presence was evaluated with logistic regression analysis.

Statistical analysis was performed in Statistica 13.1 software and Excel. Two-tailed *p*-value less than 0.05 was deemed significant.

## Results

A total of 47 patients (33 female) were included in the study. Patient characteristics are listed in Table 1. Thirty-six patients presented with IAA with VSD (76.59%), and 11 patients had CoA with VSD (23.4%). The overall incidence of chromosomal abnormalities was 31.9%, including 22q11.2 deletion syndrome in 11 patients (23.4%), CHARGE association and 5p deletion syndrome in single cases.

**Table 1:** Overall summary of the patients and LVOTO group comparison

	All patients (*n* = 47)	With LVOTO (*n* = 5)	Without LVOTO (*n* = 42)	*p*-value
Bicuspid aortic valve	47.2% (17)	80.0% (4)	41.9% (13)	0.1136^a^
Tricuspid aortic valve	52.8% (19)	20.0% (1)	58.1% (18)	
IAA	28.9% (15)	80.0% (4)	63.3% (28)	0.5454^a^
IAA A	16.2% (5)	0% (0)	17.9% (5)	0.5717^a^
IAA B	70.3% (22)	100% (4)	64.2% (18)	
IAA C	13.5% (5)	0% (0)	17.9% (5)	
CoA	31.9% (15)	20% (1)	33.3% (14)	0.5454^a^
Age at operation: Mean	23.6 (± 14.9)	20.6 (± 9.9)	24.0 (± 15.4)	0.6365^b^
Body weight at operation: Mean	3.18 (± 0.61)	3.16 (± 0.63)	3.34 (± 0.48)	0.5491^b^
BSA: Mean	0.20 (± 0.02)	0.22 (± 0.02)	0.20 (± 0.02)	0.0358^b^
AoV diameter: Mean	6.1 (± 1.4)	5.2 (± 1.4)	6.3 (± 1.4)	0.1122^b^
AV z-score: Mean	− 1.7 (± 1.7)	− 3.6 (± 2.0)	− 1.4 (± 1.5)	0.0064^b^
MV diameter: Mean	9.9 (± 2.2)	8.6 (± 1.3)	10.1 (± 2.3)	0.2222^b^
MV z-score: Mean	− 0.66 (± 1.32)	− 1.30 (± 0.89)	− 0.55 (± 1.37)	0.3080^b^
ECC [min]: Median	102 (78–118)	105 (47.5–116)	102 (78–123)	0.7921^c^
XCT [min]: Mean	53.2 (± 12.8)	52.8 (± 11.8)	53.3 (± 13.0)	0.9419^b^
ECC stop [min]: Median	24 (22–29)	28.5 (24.5–29.5)	24 (22–28)	0.3778^c^

*LVOTO* left ventricular outflow tract obstruction, *IAA* interrupted aortic arch, *CoA* coarctation of the aorta, *BSA* body surface area, *AoV* aortic valve, *MV* mitral valve, *ECC* extracorporeal circulation, *XCT* aortic cross-clamping time, *ECC stop* end of extracorporeal circulation

^a^Chi-squared test with Yates’ correction for continuity

^b^Students’ *t*-test

^c^U Mann–Whitney test

The study group (47 patients) underwent one-stage repair through median sternotomy: resection with end-to-end anastomosis, either with or without extension. The median age at the initial surgery was 23 days (range of 14–27). Three patients died within 5 years after the primary repair. One patient died of heart failure 2 days after the primary repair, one died 1.5 months after the primary repair, and one died 6 days after Ross-Konno procedure.

In the median follow-up of 8.31 years (range of 6.15–10.27) 5 patients (10.6%) presented with a significant LVOTO requiring re-intervention. Among the other 42 patients, the left ventricle outflow tract remained unobstructed. Preoperatively, the mean AoV z-score was significantly smaller in those who developed LVOTO as compared to those who did not (− 3.58 ± 1.96 vs. − 1.44 ± 1.55; *p* = 0.0016).

### Aortic Valve Growth

**Fig. 1:**
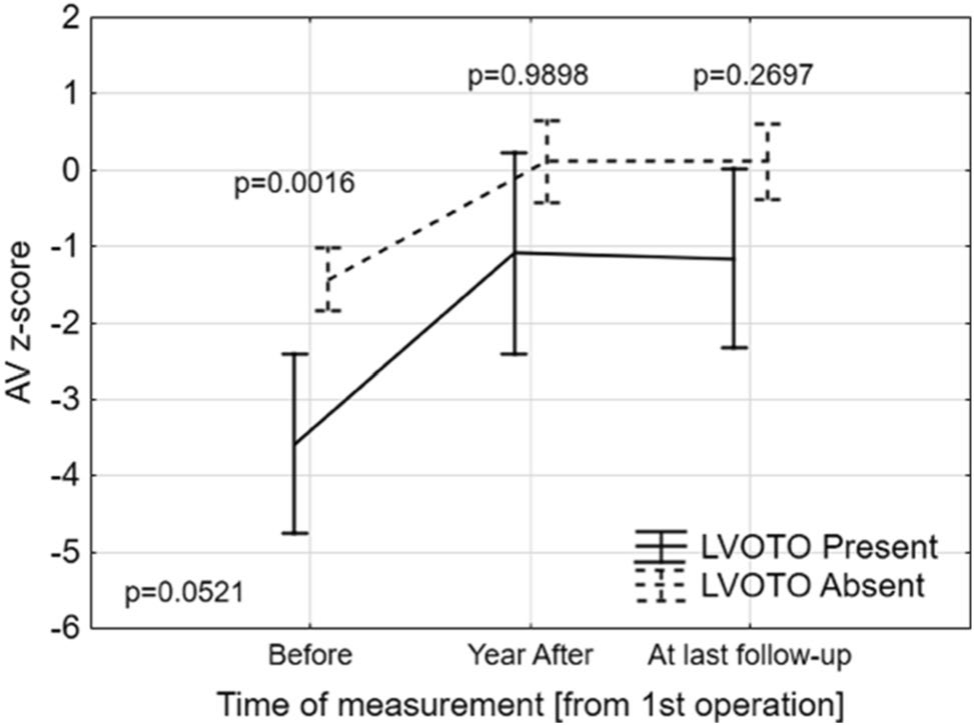
Comparison of AV z-scores before, one year after operation and at last follow-up. ANOVA for repeated measure *p*-value was 0.0521. For each time-point, Fishers’ LSD post-hoc *p*-value was: before *p* = 0.0016, one year after *p* = 0.9898, at last follow-up *p* = 0.2697

The AoV size before the initial surgical repair, one year after final repair, and at last follow-up were evaluated. Results are presented in Fig. 1 and in Supplementary Table 1. At 1-year follow-up, both groups showed an increase in the AoV size, which had z-scores that approached 0 (*p* < 0.01). In the group of patients with LVOTO the AoV z-score changed from − 3.58 ± 1.96 to an AoV z-score of − 1.09 ± 1.05, and in patients who did not develop LVOTO changed from − 1.44 ± 1.55 to 0.11 ± 1.09 (*p* = 0.98). At last follow-up, the mean AoV z-score of the patients with LVOTO was − 1.16 ± 1.40, whereas the mean AoV z-score of the other group was 0.11 ± 0.99 (*p* = 0.27).

### Risk Factors for LVOTO After Primary Repair

Multivariable logistic regression identified AoV z-score (OR 0.44, *p* = 0.017) as a predictor of LVOTO (Table 2). There were no significant differences between those two groups with regard to age, body weight at operation, mitral valve z-score, morphology of the AoV (bicuspid/tricuspid), and type of VSD (Table 2).

**Table 2:** Risk factors for LVOTO, from logistic regression model

	OR	*p*-value
Age at operation	0.980 (0.902–1.065)	0.6320
Body mass at operation	1.589 (0.360–7.025)	0.5412
AoV z-score	0.437 (0.221–0.864)	0.0173
MV z-score	0.661 (0.300–1.456)	0.3040
BV/TV (TV)	0.181 (0.018–1.809)	0.1454
VSD type		
1	0.248 (0.056–1.154)	0.0629
2	–	0.9972
3	0.250 (0.053–1.177)	0.0795

*OR* odds ratio, *AoV* aortic valve, *MV* mitral valve, *BV* bicuspid aortic valve, *TV* tricuspid aortic valve, *VSD* ventricular septal defect, *VSD type 1* conoventricular septal defect, *VSD type 2* muscular ventricular septal defect, *VSD type 3* membranous ventricular septal defect

A detailed description of LVOTO type, AV z-score, mean LVOT pressure gradient by echo, and interventions performed in patients with LVOTO are presented in the Table 3. The mean AV z-score in five patients with LVOTO was − 3.6 ± 2.0 at the time of diagnosis. Three patients with LVOTO (3/5, 60%) had AoV z-score less than − 3.0 at the time of diagnosis. Among them, one had the smallest AoV z-score of − 6.09. Similarly, 4 patients had bicuspid AoV, whereas 1 patient had tricuspid AoV. Four patients (4/5, 80%) with LVOTO required reoperation a median of 2.3 years (range of 0.3–7.9) after the initial surgery. One patient underwent stent implantation in the aortic arch at the age of 9.9 years and did not require any surgical treatment.

**Table 3:** Profiles of the patients with LVOTO (*n* = 5)—level of obstruction, AoV z-score, mean PG, and type of intervention

	Level of obstruction	AoV z-score	Mean PG [mmHg]	Age at the intervention [years]
Pt 1	Subvalvular	− 3.90	120	aortic arch plasty (0.3) sub-AS resection (3.3) Ross-Konno procedure (10.3)
Pt 2	Sub- and valvular	− 4.15	37	stent implantation in the aortic arch (9.9) observation
Pt 3	Sub- and valvular	− 4.38	88	sub-AS resection (1.6) Ross-Konno procedure (5)
Pt 4	Subvalvular	− 0.8	60	sub-AS resection (7.9)
Pt 5	Subvalvular	− 2.80	51	BVP (0.1) sub-AS resection (2)

*AS* aortic stenosis, *AoV* aortic valve, *PG* pressure gradient, *BVP* balloon valvuloplasty

The main reason for surgical re-intervention was LVOTO with an estimated echocardiographic peak gradient ≥ 60 mmHg (three patients); two patients had an estimated peak gradient of 37 and 51 mmHg. One of them underwent surgical treatment because of impaired LV function, the other underwent stent implantation in the aortic arch.

Surgical treatment consisted of resection of subaortic membrane in four patients and modified Ross-Konno procedure in two patients. Additionally, one patient underwent re-operation in the aortic arch.

### Postoperative Course After Primary One-Stage Repair

**Fig. 2:**
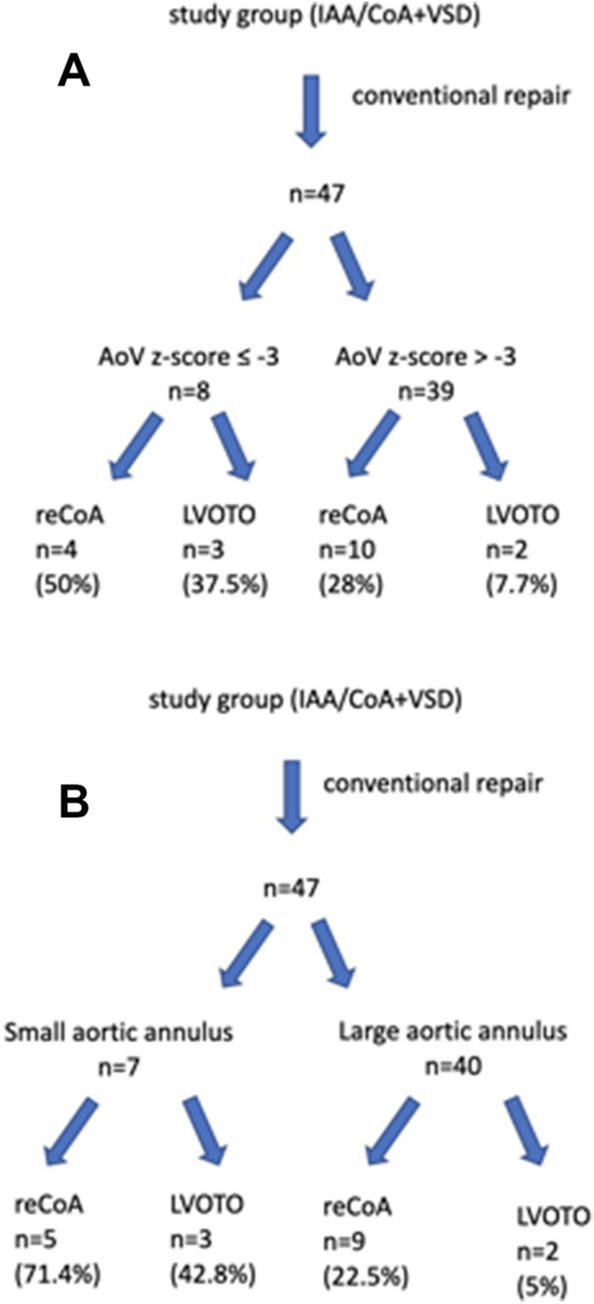
Flow charts of follow-up process of patients after conventional repair for IAA/CoA with VSD. Patients are classified to the subgroup defined by AoV z-score (**A**) or Hirata classification (**B**). *AoV* aortic valve, *reCoA* recoarctation of the aorta, *LVOTO* left ventricular outflow tract obstruction

**Fig. 3:**
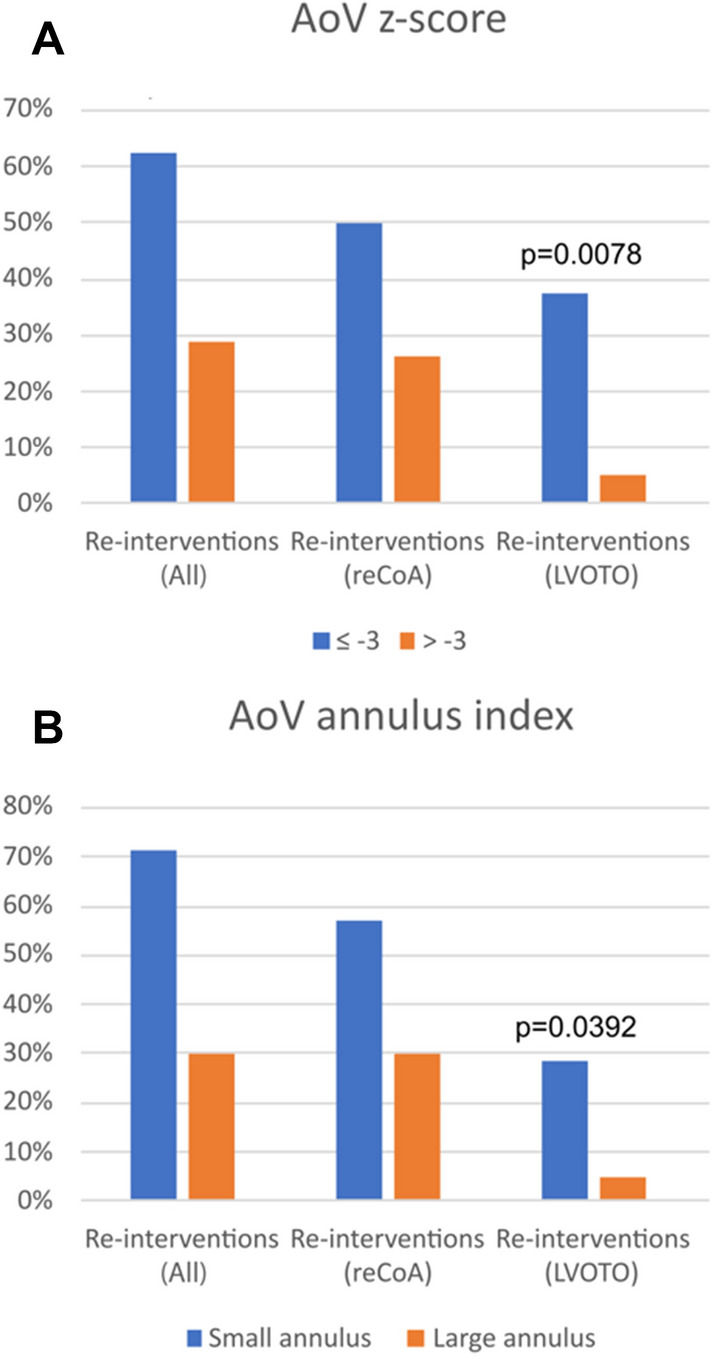
Number of patients classified to the subgroup defined by AoV z-score (**A**) or Hirata classification (**B**), based on specific reoperation/intervention. *AoV* aortic valve, *reCoA* recoarctation of the aorta, *LVOTO* left ventricular outflow tract obstruction

All surgical re-operations performed in the entire study group involved subaortic resection and redo CoA repair, whereas all percutaneous re-interventions included aorta balloon angioplasty and arch stenting. Figures 2, 3 and Supplementary Table 2 display re-intervention rate in the entire cohort, both re-operations and percutaneous interventions, separated by AoV z-score and AoV index introduced by Hirata et al. We identified 7 patients (14.9%) with small aortic annulus and 40 patients (85.1%) with large aortic annulus. According to the second classification we identified 8 patients (17.7%) with the AoV annulus z-score of − 3.0 or less and 39 patients (82.3%) with the AoV annulus z-score of ≥  − 3.0.

The re-intervention rate for LVOTO was significantly higher in patients with initial AoV z-score ≤  − 3 (37.5% vs. 7.7%; *p* = 0.008). The re-intervention rate for reCoA was higher (but not significant) in patients with initial AoV z-score ≤  − 3 (28.2% vs. 50%; *p* = 0.46). Similarly, there was significantly higher incidence of LVOTO in patients, whose aortic annulus was ≤ patient’s weight (kg) + 1.5 mm as compared to those with larger AoV annulus (42.8% vs. 5.0%; *p* = 0.04). The rate for re-intervention for reCoA was also higher in the group of patients with small aortic as compared to those with larger AoV (57.1% vs. 30.0%; *p* = 0.16), but with no significant difference.

## Discussion

In this study, we analyzed the mid- to long-term outcomes after conventional repair for patients with IAA/CoA with VSD, focusing on risk factors for post-operative left ventricular outflow tract obstruction. Postoperative LVOTO is considered as a common source of morbidity and mortality after primary repair for IAA or CoA [3, 4, 6, 7]. Our cohort included 5 patients who developed subaortic stenosis and among them 4 needed re-operation. After analysis of all the variables in our patients, the aortic valve z-score at diagnosis could be identified as a risk factor for future LVOTO. We found that the initial mean AoV z-score in patients who did not have recurrent LVOT obstruction was − 1.44 ± 1.55.

Several previous reports have tried to identify parameters used to predict the development of LVOT obstruction after IAA/CoA + VSD repair [3, 4, 8–15]. As stressed by many authors careful preoperative planning may identify high-risk patients, help to guide post-operative management, minimize the need for LVOT reintervention, and improve long-term survival [7, 13, 16, 17].

The measurement of the aortic valve annulus has been used to predict postoperative LVOTO by Hirata et al. [8]. They reported that for the patients whose aortic annulus is greater than patient’s weight + 1.5 mm, low reoperation rate for LVOTO is expected. For the patient whose aortic annulus is less than patient’s weight + 1.5 mm, almost half of them needed reoperation. The authors recommended the Yasui or the Norwood operation for patients whose aortic annulus was less than or equal to the patient’s weight + 1.0 mm.

Sugimoto et al. investigated the impact of bicuspid aortic valve on the mid- to long-term aortic valve-related outcomes after conventional repair for 50 patients with IAA/CoA combined with VSD [9]. They found bicuspid aortic valve to be a significant risk factor for valve-related reinterventions after conventional repair for IAA/COA with VSD. According to the authors careful follow-up focusing on AoV function is important also in patients who had no valve-related problems during the postoperative period.

In another series of 75 patients with IAA/CoA + VSD a bicuspid aortic valve and an aortic valve annular z-score of − 3.0 or less before primary repair were found to be risk factors for LVOTO [10]. Furthermore, bicuspid AoV patients more frequently required reoperation than tricuspid AoV patients. This finding underlines the impact of bicuspid AoV and need for case-by-case evaluation.

Salem et al. demonstrated that the most important independent predictor of subsequent LVOT obstruction in patients with IAA + VSD is AoV annulus size: AoV valve annulus diameter < 4.5 mm and a z-score < –5.0 predict development of LVOT obstruction and possible need for future surgical intervention [11]. An adequate growth of the AoV annulus seen in their whole study group is consistent with the results of our study. The authors also emphasized the role of careful monitoring not only short- and intermediate-term results of surgical repair, but also the long-term changes in the pathophysiology of this disease proces.

The minimum size of the subaortic area below which patients undergoing one-stage repair might be at high risk for postoperative LVOTO has also been controversial. Geva et al. [13] found that among a variety of anatomic risk factors a subaortic cross-sectional area ≤ 0.7 cm^2^/m^2^ was a predictor of postoperative LVOTO after repair of IAA.

Similar results reported Apfel et al. [3]. They found that those patients with significant LVOTO after conventional repair had significantly smaller subaortic diameter indexes (0.83 ± 0.1 cm/[BSA]) when compared with those with a good result (0.99 ± 0.16 cm/[BSA]).

Recently, Jijeh et al. analyzed the growth and predictors of future obstruction of the LVOT after repair for VSD and aortic obstruction [18]. They reported that small aortic valve and LVOT at diagnosis are not risk factors to predict the need for surgical re-intervention for LVOTO in future. This finding is inconsistent with a number of previous reports and the current study as well.

Selective management of the LVOT for repair of IAA with VSD was reported by Suzuki et al. [12]. They demonstrated that, tailored to the degree of subaortic narrowing, resection or incision of the infundibular septum at the time of primary repair was very effective in preventing or prolonging the interval to recurrent LVOTO. However, they found that reoperation for LVOTO related to the development of a new and discrete subaortic membrane or aortic stenosis is still required in a subset of patients. Many authors report that prophylactic direct approaches to prevent future LVOT obstruction including myectomy/myotomy and left-sided placement of the VSD patch do not reliably prevent late LVOTO. It is unclear if they are effective in preventing postrepair LVOTO compared with a conventional repair [16].

One of the major findings of our study is that aortic annuluses grew significantly within 1 year after primary repair. In particular, the aortic annulus of patients, who developed postoperative LVOTO, had increased in size from mean z-score of − 3.58 to mean z-score of − 1.09 at one year after the primary repair. The growth of the annular dimension, in response to increased flow after repair, has been reported in previous reports. The above mentioned study of 75 patients by Sugiura et al., and similarly by Sugimoto et al. [9], found that regardless of the annular size before primary repair, the size of the aortic valve annulus increased in almost all patients after surgery [10]. In both groups (bicuspid/tricuspid aortic valve) the aortic valve diameter became significantly larger at the 1-year follow-up, approximating the normal value. Significant LVOT growth after repair of aortic obstruction with VSD in 89 patients was observed also by Jijeh et al. [18]. The recent study of Plymale et al. found normalization of the aortic valve z-score at follow-up, but residual aortic arch obstruction persisted in one-third of subjects [19].

Our study, similarly to others, has shown that the aortic valve annulus grows after initial repair and establishment of increased aortic flow, but it is still not enough to prevent post-operative LVOTO if the initial aortic valve z-score was − 3.6 ± 2.0.

In the current study, we found increased re-intervention rate, either for LVOTO or reCoA, in the group of patients with small aortic annulus during long-term follow-up.

Previous studies have shown that apart from hypertension and re-coarctation, aortic valve and aortic arch pathology are commonly encountered in patients after coarctation repair [20–22].

That indicates need for case-by-case follow-up evaluation of patients with CoA/IAA, especially those with small aortic valve before primary repair.

There are several limitations to this retrospective and single-institution study including relatively small-sized cohort and the small number of events. The rates for re-intervention for reCoA, although higher in the group of patients with small aortic annulus and AoV z-score of ≤  − 3, did not meet significance, which is most likely due to a small sample size. A longer follow-up period is required to confirm the results of the current study.

## Conclusion

In patients after surgical treatment of IAA/CoA with VSD, the AoV z-score at the time of diagnosis is a significant risk factor for reoperation for LVOTO. With age, AoV growth and z-score improvement is expected, with the most growth in the first year after primary repair. Small AoV dimension is correlated with increased rate of re-operations and catheter re-interventions for LVOTO and recoarctation of the aorta. Despite good overall survival, patients after conventional repair for IAA/CoA with VSD frequently require reintervention and need to be carefully followed.

## Supplementary Information

Below is the link to the electronic supplementary material.

Supplementary file1 (DOCX 55 kb)

Supplementary file2 (DOCX 56 kb)

## References

[1] Schreiber C, Mazzitelli D, Haehnel JC, Lorenz HP, Meisner H (1997). The interrupted aortic arch: an overview after 20 years of surgical treatment. Eur J Cardiothorac Surg.

[2] Brown JW, Ruzmetov M, Okada Y, Vijay P, Rodefeld MD, Turrentine MW (2006). Outcomes in patients with interrupted aortic arch and associated anomalies: a 20-year experience. Eur J Cardiothorac Surg.

[3] Apfel HD, Levenbraun J, Quaegebeur JM, Allan LD (1998). Usefulness of preoperative echocardiography in predicting left ventricular outflow obstruction after primary repair of interrupted aortic arch with ventricular septal defect. Am J Cardiol.

[4] Chen PC, Cubberley AT, Reyes K (2013). Predictors of reintervention after repair of interrupted aortic arch with ventricular septal defect. Ann Thorac Surg.

[5] Pettersen MD, Du W, Skeens ME, Humes RA (2008). Regression equations for calculation of z scores of cardiac structures in a large cohort of healthy infants, children, and adolescents: an echocardiographic study. J Am Soc Echocardiogr.

[6] Kreutzer J, Van Praagh R (2000). Comparison of left ventricular outflow tract obstruction in interruption of the aortic arch and in coarctation of the aorta, with diagnostic, developmental, and surgical implications. Am J Cardiol.

[7] Jegatheeswaran A, McCrindle BW, Blackstone EH (2010). Persistent risk of subsequent procedures and mortality in patients after interrupted aortic arch repair: a Congenital Heart Surgeons' Society study. J Thorac Cardiovasc Surg.

[8] Hirata Y, Quaegebeur JM, Mosca RS, Takayama H, Chen JM (2010). Impact of aortic annular size on rate of reoperation for left ventricular outflow tract obstruction after repair of interrupted aortic arch and ventricular septal defect. Ann Thorac Surg.

[9] Sugimoto A, Ota N, Miyakoshi C (2014). Mid- to long-term aortic valve-related outcomes after conventional repair for patients with interrupted aortic arch or coarctation of the aorta, combined with ventricular septal defect: the impact of bicuspid aortic valve. Eur J Cardiothorac Surg.

[10] Sugiura J, Nakano T, Kado H (2016). Left ventricular outflow tract obstruction in aortic arch anomalies with ventricular septal defect. Ann Thorac Surg.

[11] Salem MM, Starnes VA, Wells WJ (2000). Predictors of left ventricular outflow obstruction following single-stage repair of interrupted aortic arch and ventricular septal defect. Am J Cardiol.

[12] Suzuki T, Ohye RG, Devaney EJ (2006). Selective management of the left ventricular outflow tract for repair of interrupted aortic arch with ventricular septal defect: management of left ventricular outflow tract obstruction. J Thorac Cardiovasc Surg.

[13] Geva T, Hornberger LK, Sanders SP, Jonas RA, Ott DA, Colan SD (1993). Echocardiographic predictors of left ventricular outflow tract obstruction after repair of interrupted aortic arch. J Am Coll Cardiol.

[14] Oosterhof T, Azakie A, Freedom RM, Williams WG, McCrindle BW (2004). Associated factors and trends in outcomes of interrupted aortic arch. Ann Thorac Surg.

[15] Minich LL, Snider AR, Bove EL, Lupinetti FM (1992). Echocardio- graphic predictors of the need for infundibular wedge resection in infants with aortic arch obstruction, ventricular septal defect and subaortic stenosis. Am J Cardiol.

[16] Riggs KW, Tweddell JS (2019). How small is too small? Decision-making and management of the small aortic root in the setting of interrupted aortic. Arch Semin Thorac Cardiovasc Surg Pediatr Card Surg Annu.

[17] Abarbanell G, Border WL, Schlosser B, Morrow G, Kelleman M, Sachdeva R (2018). Preoperative echocardiographic measures in interrupted aortic arch: which ones best predict surgical approach and outcome?. Congenit Heart Dis.

[18] Jijeh A, Ismail M, Alhabshan F (2017). Growth of left ventricular outflow tract and predictors of future re-intervention after repair for ventricular septal defect and aortic arch obstruction. Cardiol Young.

[19] Plymale JM, Frommelt PC, Nugent M (2017). The infant with aortic arch hypoplasia and small left heart structures: echocardiographic indices of mitral and aortic hypoplasia predicting successful biventricular repair. Pediatr Cardiol.

[20] Roos-Hesselink JW, Schölzel BE, Heijdra RJ (2003). Aortic valve and aortic arch pathology after coarctation repair. Heart.

[21] Luijendijk P, Stevens AW, de Bruin-Bon RH (2013). Rates and determinants of progressive aortic valve dysfunction in aortic coarctation. Int J Cardiol.

[22] Keshavarz-Motamed Z, Garcia J, Kadem L (2013). Fluid dynamics of coarctation of the aorta and effect of bicuspid aortic valve. PLoS ONE.

