# Supplementary material for: Predictors of Left Ventricular Outflow Tract Obstruction After Conventional Repair for Patients with Interrupted Aortic Arch or Coarctation of the Aorta, Combined with Ventricular Septal Defect: A Single-Center Experience
Source: Pediatr Cardiol. 2021 Oct 26;43(3):525–31. doi: 10.1007/s00246-021-02749-0 (PMC8933342; doi:10.1007/s00246-021-02749-0)
Supplement: Supplementary file 1 — Supplementary file1 (DOCX 55 kb) [file 246_2021_2749_MOESM1_ESM.docx]

**Supplementary Table 1:** Mean and standard deviation for AoV z-score before, one year after and at last follow-up for all patients and LVOTO groups.

| AoV  z-score | All patients (n=47) | | | With LVOTO (n=5) | | | Without LVOTO (n=42) | | |
| --- | --- | --- | --- | --- | --- | --- | --- | --- | --- |
|  | **Before** | **One year after** | **At last follow-up** | **Before** | **One year after** | **At last follow-up** | **Before** | **One year after** | **At last follow-up** |
| Mean | -1.67 | -0.06 | -0.09 | -3.58 | -1.09 | -1.16 | -1.44 | 0.11 | 0.11 |
| SD | 1.71 | 1.15 | 1.13 | 1.96 | 1.05 | 1.40 | 1.55 | 1.09 | 0.99 |
| Mean-SD | -3.38 | -1.21 | -1.22 | -5.54 | -2.14 | -2.56 | -2.99 | -0.98 | -0.88 |
| Mean+SD | 0.04 | 1.09 | 1.04 | -1.62 | -0.04 | 0.24 | 0.11 | 1.20 | 1.10 |

AoV – aortic valve, LVOTO – left ventricular outflow tract obstruction, SD – standard deviation
