# Supplementary material for: Predictors of Left Ventricular Outflow Tract Obstruction After Conventional Repair for Patients with Interrupted Aortic Arch or Coarctation of the Aorta, Combined with Ventricular Septal Defect: A Single-Center Experience
Source: Pediatr Cardiol. 2021 Oct 26;43(3):525–31. doi: 10.1007/s00246-021-02749-0 (PMC8933342; doi:10.1007/s00246-021-02749-0)
Supplement: Supplementary file 2 — Supplementary file2 (DOCX 56 kb) [file 246_2021_2749_MOESM2_ESM.docx]

**Supplementary Table 2.** Number of patients classified to the subgroup defined by AoV z-score or Hirata classification, based on specific reoperation/intervention.

|  | **AoV z-score** | | | **Aortic annulus index** | | |
| --- | --- | --- | --- | --- | --- | --- |
|  | ≤ -3  (8) | > -3  (39) | p-value | Small annulus  (7) | Large annulus (40) | p-value |
| **Re-interventions** | 5 (62.5%) | 11 (28.2%) | 0.0701^1^ | 5 (71.4%) | 12 (30.0%) | 0.0353^1^ |
| **Re-interventions (reCoA)** | 4 (50.0%) | 10 (25.6%) | 0.4648^1^ | 5 (71.4%) | 9 (22.5%) | 0.1621^1^ |
| **Re-interventions (LVOTO)** | 3 (37.5%) | 2 (5.1%) | 0.0078^1^ | 3 (42.8%) | 2 (5.0%) | 0.0392^1^ |

1 – Fisher’s exact test, 2 – Chi-squared test with Yates’ correction for continuity. AoV – aortic valve, reCoA - recoarctation of the aorta, LVOTO - left ventricular outflow tract obstruction
